# Factors associated with local breast cancer recurrence after mastectomy in the Netherlands: a retrospective nationwide cohort study

**DOI:** 10.1016/j.breast.2026.104844

**Published:** 2026-06-15

**Authors:** T.D.A. Stindt, M.C. van Maaren, B.V. Offersen, S. Siesling, D.J.P. van Uden, P.M.P. Poortmans, L.J. Boersma, O. Kaidar-Person

**Affiliations:** a Department of Health Technology and Services Research, Technical Medical Centre, University of Twente, P.O. Box 217, Enschede, 7500 AE, the Netherlands; b Department of Research and Development, Netherlands Comprehensive Cancer Organisation (IKNL), Utrecht, the Netherlands; c Department of Experimental Clinical Oncology, Aarhus University Hospital, Denmark; d Department of Surgery, Canisius Wilhelmina Hospital, Nijmegen, the Netherlands; e Department of Radiation Oncology, Iridium Netwerk, Wilrijk-Antwerp, Belgium; f Faculty of Medicine and Health Sciences, University of Antwerp, Wilrijk-Antwerp, Belgium; g Department of Radiation Oncology (Maastro), GROW- Research Institute for Oncology and Developmental Biology, Maastricht University, Maastricht, the Netherlands; h Gray School of Medical Sciences, Faculty of Medical and Health Science, Aviv University, Tel-Aviv, Israel; i Breast Radiation Unit, Chaim Sheba Medical Center, Tel-Hashomer, Ramat-Gan, Israel

**Keywords:** Breast cancer, Local recurrence, Mastectomy, Risk factors, Primary systemic therapy, Radiation therapy

## Abstract

**Background:**

Despite presumed complete removal of glandular breast tissue, breast cancer (BC) patients can experience local recurrence (LR) after mastectomy. Most trials report recurrences as locoregional recurrence, potentially obscuring risk factors for LR. In this study, incidence of and risk factors for isolated LR after mastectomy were analysed.

**Methods:**

Women diagnosed with primary unilateral nonmetastatic BC treated with mastectomy between 2012 and 2016 were selected from the Netherlands Cancer Registry. LR incidence was assessed within guideline-based indication groups. Multivariable Cox regression models were constructed for patients with and without primary systemic therapy (PST).

**Results:**

In total, 17,082 non-PST and 5033 PST patients were included, of whom 345 (2%) and 80 (1.6%) had LR after a median follow-up of 7.8 and 6.7 years, respectively. LR rates were low but varied between indication groups. In the non-PST group, screening detection, low grade, targeted therapy while HER2-positive, radiation therapy (RT), and adjuvant chemotherapy were associated with lower LR risk. Younger age, inner-quadrant location, higher tumour and nodal stage, immediate reconstruction, and omission of endocrine therapy were associated with higher LR risk. In the PST group, pathologic complete response, HER2-positivity, and RT were associated with lower LR risks, while higher clinical T stage, presence of ductal carcinoma in situ, and no endocrine therapy while hormone-receptor positive were associated with higher LR risk.

**Conclusion:**

We identified several factors associated with LR rates after mastectomy. Despite the observational character of our study, these factors could be considered in future treatment decision-making.

## Highlights

• Local recurrence rates after mastectomy are generally low.
• Multiple factors associated with local recurrence after mastectomy were identified.
• Risk factors differed between patients with and without primary systemic therapy.
• Results support personalised treatment decisions before and after mastectomy.

## 1 Introduction

In recent decades, the incidence of breast cancer has risen. In 2023, over 15,000 women were diagnosed with invasive breast cancer in the Netherlands [1]. Around 30% of patients with early breast cancer are treated by mastectomy with or without post-mastectomy radiation therapy (PMRT) [2]. After curative treatment, breast cancer survivors have a 10-year risk of developing local (4.7%), regional (3%), or distant recurrences (15%) [3]. However, few studies specifically investigate local recurrence (LR) after mastectomy, and its risk factors remain poorly understood. Researchers often group LR with regional recurrence as locoregional recurrence (LRR) without distinguishing between the two[[4], [5], [6]], possibly due to the limited number of events. This restricts further advancement in the personalised treatment of breast cancer patients. To address this knowledge gap, this study aims to determine factors associated with the occurrence of LR only, without regional recurrence, in patients with primary invasive nonmetastatic breast cancer after mastectomy in the Netherlands.

## 2 Methods

### 2.1 Data source and study population

Data used in this nationwide retrospective cohort study were obtained from the Netherlands Cancer Registry (NCR), hosted by the Netherlands Comprehensive Cancer Organisation (IKNL). This registry consists of population-based data of all cancer patients in the Netherlands diagnosed since 1989. All women diagnosed with their first primary invasive nonmetastatic breast cancer between 2012 and 2016 who underwent mastectomy were selected. Patients treated in hospitals outside of the Netherlands or with synchronous breast cancer, defined as a second primary tumour within 90 days, were excluded. Patients who were male, whose diagnosis was an incidental finding (for example, during breast augmentation), had positive resection margins after mastectomy, unknown mastectomy dates, incomplete follow-up, or previous breast surgery for oncological reasons were also excluded.

The study population was divided into two subgroups based on whether patients had received primary systemic therapy (PST), *i.e.,* neoadjuvant systemic therapy, as these groups differ in prognostic factors and are not directly comparable. The non-PST group consisted of patients who underwent primary mastectomy at the time of breast cancer diagnosis, followed by adjuvant systemic therapy, *i.e.,* chemotherapy, endocrine therapy or targeted therapy or combinations, or no adjuvant therapy, based on patient- and tumour-related risk factors as indicated in the Dutch guidelines (2012). The PST group consisted of patients who received PST before mastectomy, with and without adjuvant systemic therapy after mastectomy. According to the Dutch guidelines (2012), PST is recommended for locoregionally extensive (stage III) breast cancer and may be considered in stage II disease when systemic therapy is indicated at diagnosis based on molecular subtype, *e.g.*, triple negative, or when tumour downsizing is desirable, for example, to facilitate breast-conserving therapy (Supplementary Table 1). As only invasive breast cancer was considered and tumour (T) and nodal (N) stage were key prognostic factors, non-PST patients with pT0 or unknown pT or pN and PST patients with cT0, cTis or unknown cT or cN were excluded. Additionally, PST patients who received only preoperative RT or targeted therapy without chemotherapy were excluded.

### 2.2 Data collection on recurrence

Patient data were collected from the NCR using a linkage with the Dutch Nationwide Pathology Databank (https://www.palga.nl/en_GB/data/public-database). An algorithm based on diagnostic codes (including tumour topography), free text, and date of diagnosis generated notifications indicating whether a patient was suspected of an LRR. For patients with a notification, data on LRR (if applicable) were collected by manually reviewing patient files and selecting patients with only an LR. Patient files without a notification were assumed to be free of pathologically diagnosed LRR (irrespective of the presence of any potential distant metastasis) and not searched. Completeness of LRR is estimated to be around 80% (meaning that we miss about 20% of the LRR), based on a validation study including patients diagnosed between January and March 2012, for whom data were collected using the standard search of all patient files and the notification method [7]. The information on LRR is complete until November 2022.

### 2.3 Outcome and definitions

The primary outcome was post-mastectomy LR, defined as any epithelial breast cancer or ductal carcinoma in situ (DCIS) in the ipsilateral chest wall skin or subcutaneous tissue [4]. Follow-up was calculated from the date of mastectomy to the occurrence of an event, *i.e.,* LR or competing event or last observation. Competing events were regional recurrence, distant metastasis, contralateral breast event, or death. In case of multiple events, the first event was considered. If LR occurred within 30 days of a competing event, LR was assumed to pre-exist the competing event and used as the primary endpoint. Hormone receptor positivity was defined as either oestrogen receptor or progesterone receptor positivity, *i.e.*, ≥ 10% nuclear staining. For the PST group, pathologic complete response (pCR) was defined as ypT0N0 or ypTisN0 [8].

Guideline-based indication groups were defined by pathological and clinical stage and four predefined risk factors, namely malignancy grade 3, triple-negative subtype, age ≤40 years, and tumour size >3 cm. These criteria reflect the indications for PST and RT in the 2012 Dutch national breast cancer guidelines. In the non-PST group, the indication groups were pT1–2N0 with ≥3 risk factors; pT1–2N1 or pT3N0 with ≥1 risk factor; and pT4N0, pT3N+, or ≥ pN2 disease. In the first two indication groups, the guidelines advised discussing the pros and cons of chest wall RT with the patient; in the latter, RT was recommended. For the PST group, the indication groups were cN2-3 disease; cT3N0 with ≥1 risk factor; cT4 disease; cT1–2N0 with ≥3 risk factors and no pCR; and cT1–2N+ with ≥1 risk factor and no pCR; and ypN1 or higher.

### 2.4 Statistical analysis

Patient-, tumour-, and treatment-related characteristics were summarised using descriptive statistics. LR incidence was calculated for the guideline-based indication groups. Associations with LR were first assessed using univariable Cox proportional hazards regression. The proportional hazards assumption was evaluated by visually inspecting time independence of Schoenfeld residuals and parallelism of the log-log survival curves [9]. Variables that were differently distributed between outcome status and had significant association with the outcome in univariable Cox regression (p-value≤0.2) were included in the multivariable Cox regression model to estimate hazard ratios (HRs) with 95% confidence intervals [10]. To avoid collinearity, hormone receptor status and endocrine therapy were combined. For the same reason, human epidermal growth factor receptor 2 (HER2) status and targeted treatment were also combined in the non-PST group. This was not viable in the PST group because almost all HER2-positive patients received targeted therapy. Using backward selection, variables that did not significantly (p-value<0.05) contribute to the model according to the likelihood ratio test were removed [11]. Patients were censored at the time of competing events, following the principle of cause-specific Cox regression [12]. Missing data were considered missing at random and imputed using the ‘futuremice’ package in R [13]. The number of imputations required to produce replicable standard errors in the multivariable Cox regression model was determined using Von Hippel's method [14]. This resulted in 33 and 55 imputations for the non-PST and PST groups, respectively, with 10 and 40 iterations per imputation to achieve convergence. To accelerate computation, imputations ran in parallel on four processor cores. Composite variables, *i.e.,* hormonal status ± endocrine therapy, HER2 status ± targeted therapy, and pCR, were imputed using the ‘impute, then transform’ method [15]. Imputation validity was evaluated using convergence plots of the mean and standard deviation and by comparing the distributions of the imputed and observed data. Estimates from imputed datasets were pooled and compared with those from complete cases. Collinearity among variables in the multivariable model was evaluated using the variance inflation factor (VIF), with a threshold of 4, and potential variable interactions were evaluated [16]. Goodness of fit was evaluated by visually inspecting the Cox-Snell residuals [17]. Analyses were performed in R, version 4.4.1.

This study is part of the Spatial location of breast cancer local rECurRence aftEr masTectomy (SECRET) study, which aims to improve the definition of RT target volumes of the chest wall after mastectomy (NCT06130111) [18].

## 3 Results

### 3.1 Patient inclusion

**Fig. 1:**
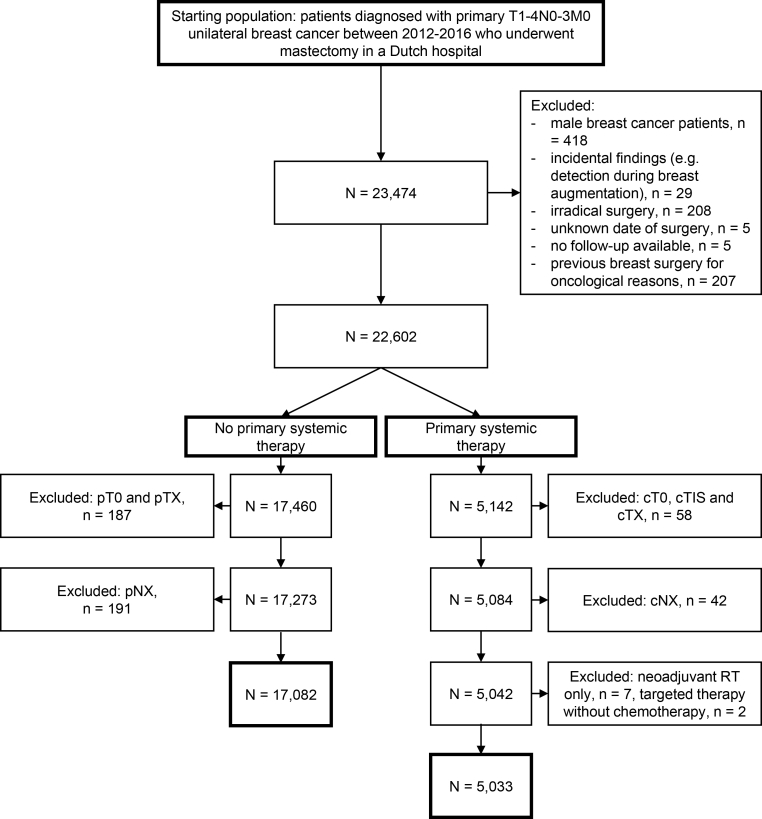
Flowchart of patient inclusion results.

A total of 22,602 patients were eligible for inclusion. After additional exclusions, 17,082 patients in the non-PST group and 5033 in the PST group were included for analysis (Fig. 1).

Both subgroups differed in terms of demographics, tumour, and treatment characteristics. Over time, more patients were treated with PST. Patients in the non-PST group were generally older, more often postmenopausal and detected by population screening. Compared with the PST group, they more often presented with smaller tumours, absence of nodal involvement, a DCIS component, hormone receptor–positive and HER2-negative disease and were less likely to have tumours located in overlapping parts of the breast or multifocal tumours. In addition, patients in the non-PST group were less often treated with axillary lymph node dissection, RT, chemotherapy, endocrine therapy, and targeted treatment (Table 1).

**Table 1:** Baseline characteristics of the study population (n = 22,115).

	No primary systemic therapy (N = 17082)	Primary systemic therapy (N = 5033)
**Year of diagnosis**		
2012	3932 (23.0%)	784 (15.6%)
2013	3724 (21.8%)	892 (17.7%)
2014	3457 (20.2%)	1021 (20.3%)
2015	3202 (18.7%)	1181 (23.5%)
2016	2767 (16.2%)	1155 (22.9%)
**Age**		
<40	935 (5.5%)	871 (17.3%)
40-49	2872 (16.8%)	1645 (32.7%)
50-59	3818 (22.4%)	1209 (24.0%)
60-69	3920 (22.9%)	856 (17.0%)
70-79	3260 (19.1%)	283 (5.6%)
>79	2277 (13.3%)	169 (3.4%)
**Menopausal status at diagnosis**		
Premenopausal	3256 (19.1%)	2111 (41.9%)
Perimenopausal^a^	792 (4.6%)	309 (6.1%)
Postmenopausal	11472 (67.2%)	2025 (40.2%)
Unknown	1562 (9.1%)	588 (11.7%)
**Detected after population screening**		
No/unknown	11980 (70.1%)	4468 (88.8%)
Yes	5102 (29.9%)	565 (11.2%)
**Sublocalisation of the tumour**		
Outer quadrants	7013 (41.1%)	1863 (37.0%)
Inner quadrants	2862 (16.8%)	638 (12.7%)
Central parts	1594 (9.3%)	394 (7.8%)
Overlapping lesions	5358 (31.4%)	2081 (41.3%)
Unknown	255 (1.5%)	57 (1.1%)
**Morphology**		
Ductal	12886 (75.4%)	4050 (80.5%)
Lobular	2747 (16.1%)	703 (14.0%)
Mixed ductal lobular	732 (4.3%)	140 (2.8%)
Other	717 (4.2%)	140 (2.8%)
**Differentiation grade**		
Grade 1	3045 (17.8%)	365 (7.3%)
Grade 2	8239 (48.2%)	1502 (29.8%)
Grade 3	5205 (30.5%)	1102 (21.9%)
Unknown	593 (3.5%)	2064 (41.0%)
**Multifocality**		
No	11975 (70.1%)	3090 (61.4%)
Yes	5030 (29.4%)	1881 (37.4%)
Unknown	77 (0.5%)	62 (1.2%)
**cT-stage**		
cTis	598 (3.5%)	-
cT1	7311 (42.8%)	579 (11.5%)
cT2	7268 (42.5%)	2294 (45.6%)
cT3	1146 (6.7%)	1479 (29.4%)
cT4	215 (1.3%)	681 (13.5%)
Unknown	544 (3.2%)	-
**cN-stage**		
cN0	13836 (81.0%)	1715 (34.1%)
cN1	2975 (17.4%)	2756 (54.8%)
cN2	44 (0.3%)	118 (2.3%)
cN3	50 (0.3%)	444 (8.8%)
Unknown	177 (1.0%)	-
**pT-stage**^b^		
pTis	-	211 (4.2%)
pT0	-	1008 (20.0%)
pT1	8264 (48.4%)	1571 (31.2%)
pT2	7463 (43.7%)	1417 (28.2%)
pT3	1152 (6.7%)	571 (11.3%)
pT4	203 (1.2%)	106 (2.1%)
Unknown	-	149 (3.0%)
**pN-stage**		
pN0	9567 (56.0%)	2287 (45.4%)
pN1	5547 (32.5%)	1685 (33.5%)
pN2	1222 (7.2%)	596 (11.8%)
pN3	746 (4.4%)	297 (5.9%)
Unknown	-	168 (3.3%)
**Pathologic complete response**		
No	NA	3928 (78.0%)
Yes	NA	949 (18.9%)
Unknown	NA	156 (3.1%)
**Presence of DCIS component next to invasive component**		
No	7918 (46.4%)	2990 (59.4%)
Yes	8989 (52.6%)	1907 (37.9%)
Unknown	175 (1.0%)	136 (2.7%)
**Hormonal receptor status ± endocrine therapy**		
Positive with endocrine therapy	10645 (62.3%)	3474 (69.0%)
Positive without endocrine therapy	3562 (20.9%)	188 (3.7%)
Negative	2701 (15.8%)	1329 (26.4%)
Unknown	174 (1.0%)	42 (0.8%)
**HER2 status ± targeted therapy**^c^		
Negative	14067 (82.3%)	3743 (74.4%)
Positive with targeted therapy	1657 (9.7%)	1153 (22.9%)
Positive without targeted therapy	820 (4.8%)	41 (0.8%)
Unknown	538 (3.1%)	96 (1.9%)
**Immediate breast reconstruction**		
No	13294 (77.8%)	3579 (71.1%)
Yes	3788 (22.2%)	1454 (28.9%)
**Axillary lymph node dissection**		
No	11820 (69.2%)	2570 (51.1%)
Yes	5262 (30.8%)	2463 (48.9%)
**Radiation therapy type**		
No radiation therapy	12413 (72.7%)	1659 (33.0%)
Chest wall with boost	201 (1.2%)	61 (1.2%)
Chest wall without boost	1153 (6.7%)	631 (12.5%)
Chest wall with regional nodes and boost	208 (1.2%)	277 (5.5%)
Chest wall with regional nodes without boost	2725 (16.0%)	2191 (43.5%)
Other	264 (1.5%)	54 (1.1%)
Unknown^d^	118 (0.7%)	160 (3.2%)
**Chemotherapy**		
No	10097 (59.1%)	375 (7.5%)
Pre-surgical	-	4370 (86.8%)
Post-surgical^e^	6985 (40.9%)	38 (0.8%)
Pre- and post-surgical	-	250 (4.9%)

Abbreviations: cT = clinical tumour stage, cN = clinical nodal stage, pN = pathological nodal stage, pT = pathological tumour stage, DCIS = ductal carcinoma in situ, HER2 = human epidermal growth factor receptor 2.

a Period between the last regular menstrual cycle and a year after the last menstruation.

b ypT for the PST group.

c For the PST group, HER2 status ± treatment was reduced to HER2 status positive and negative because HER2-positive patients without targeted therapy were extremely rare.

d Patients who received RT, but for whom the RT type is unknown.

e PST patients with only post-surgical chemotherapy had either endocrine or targeted therapy before surgery, or both.

### 3.2 LR incidences per indication group

In the non-PST and PST groups, patients had a median follow-up of 7.8 and 6.7 years, during which 2% and 1.6% developed LR, respectively. Across all guideline-based indication groups, the incidence of LR varied due to the small numbers of patients and LR events but was generally low (Table 2).

**Table 2:** Incidence of LR per indication group.

Indication group	Number of patients	Number of LR	LR incidence (%)
**Non-PST**			
pT1-2N0 with ≥3 RF^a^	21	2	9.5
pT1-2N1 or pT3N0 with ≥1 RF^a^	2044	56	2.7
pT4N0, pT3N+, or ≥ pN2 disease	2492	44	1.8
**PST**			
≥ ypN1 disease	2578	46	1.8
≥ cN2 disease	562	6	1.1
cT3N0 and ≥1 RF^a^	209	4	1.9
cT4 anyN disease	681	17	2.5
cT1-2N0 and ≥3 RF^a^, and no pCR	4	0	0
cT1-2N+ and ≥1 RF^a^ and no pCR	582	9	1.5

Abbreviations: PST = primary systemic therapy, pT = patholocigal tumour stage, pN = pathological nodal stage, RF = risk factor, pCR = pathologic complete response.

a Risk factors were: differentiation grade 3, triple-negative subtype, age ≤40 years, and tumour size >3 cm.

### 3.3 Risk factors for post-mastectomy LR

In the non-PST group, age group, menopausal status, screening detection, tumour sub-localisation, morphology, differentiation grade, multifocality, pathological tumour stage, pathological nodal stage, presence of DCIS, immediate reconstruction, hormonal receptor status ± treatment, HER2 status ± treatment, RT type, and chemotherapy were included in the full model (Supplementary Table 2). No significant violations of the proportional hazards assumption were found. A reduced model was generated by removing morphology, multifocality, and the presence of a DCIS component during backward selection (Table 3).

**Table 3:** Risk factors for post-mastectomy LR in non-PST patients based on the reduced multivariable Cox regression model of the imputed dataset.

Factor	Level	HR^a^	95% CI	P-value
Age	*60-69 (reference)*			
	<40	1.43	0.74 - 2.76	0.286
	40-49	0.96	0.56 - 1.65	0.892
	50-59	1.47	1.02 - 2.13	**0.041**
	70-79	1.16	0.81 - 1.65	0.408
	>79	0.88	0.6 - 1.31	0.538
Menopausal status	*Post (reference)*			
	Pre	1.03	0.68 - 1.57	0.878
	Peri	0.54	0.27 - 1.08	0.082
Screening	*No (reference)*			
	Yes	0.66	0.49 - 0.88	**0.005**
Sub-localisation	*Outer quadrants (reference)*			
	Inner quadrants	1.36	1.02 - 1.81	**0.034**
	Central parts	0.92	0.61 - 1.38	0.682
	Overlapping lesions	1.07	0.83 - 1.39	0.593
Differentiation grade	*Grade 2 (reference)*			
	Grade 1	0.67	0.48 - 0.93	**0.017**
	Grade 3	1.04	0.79 - 1.38	0.768
pT-stage	*pT1 (reference)*			
	pT2	1.46	1.12 - 1.91	**0.005**
	pT3	1.93	1.2 - 3.12	**0.007**
	pT4	2.14	0.99 - 4.6	0.053
pN-stage	*pN0 (reference)*			
	pN1	1.46	1.12 - 1.91	**0.006**
	pN2	2.38	1.43 - 3.97	**0.001**
	pN3	3.46	1.96 - 6.09	**<0.001**
Immediate reconstruction	*No (reference)*			
	Yes	1.49	1.11 - 2	**0.009**
Hormonal receptor status ± endocrine therapy	*Positive with endocrine therapy (reference)*			
	Positive without endocrine therapy	2.08	1.56 - 2.78	**<0.001**
	Negative	2.72	2.01 - 3.69	**<0.001**
HER2 status ± targeted therapy	*Negative (reference)*			
	Positive with targeted therapy	0.3	0.15 - 0.6	**0.001**
	Positive without targeted therapy	0.87	0.56 - 1.35	0.537
Radiation therapy type	*No radiation therapy (reference)*			
	Chest wall	0.21	0.1 - 0.43	**<0.001**
	Chest wall with regional nodes	0.24	0.15 - 0.39	**<0.001**
	Other	0.74	0.3 - 1.81	0.501
Chemotherapy	*No (reference)*			
	Yes, post-surgical	0.51	0.37 - 0.72	**<0.001**

Abbreviations: HR = hazard ratio, CI = confidence interval, pN = pathological nodal stage, pT = pathological tumour stage, HER2 = human epidermal growth factor receptor 2.

a HR above 1 describes a risk-increasing effect; HR below 1 describes a protective effect.

Variables that were included in the full multivariable model for the PST group were menopausal status, screening detection, sub-localisation, morphology, differentiation grade, multifocality, clinical tumour and nodal stages, pathologic complete response, presence of a DCIS component, immediate breast reconstruction, hormonal receptor status ± treatment, HER2 status, RT type, and chemotherapy (Supplementary Table 3). No significant violations of the proportional hazards assumption were found. After backward selection, differentiation grade, clinical tumour stage, pathologic complete response, presence of DCIS component, HER2 status, hormone receptor status ± treatment, and RT type remained in the reduced model (Table 4).

**Table 4:** Risk factors for post-mastectomy LR in PST patients based on the reduced multivariable Cox regression model of the imputed dataset.

Factor	Level	HR^a^	95% CI	P-value
Differentiation grade	*Grade 2 (reference)*			
	Grade 1	0.9	0.35 - 2.31	0.822
	Grade 3	1.55	0.86 - 2.79	0.139
cT	*cT2 (reference)*			
	cT1	0.92	0.4 - 2.11	0.835
	cT3	1.36	0.78 - 2.4	0.276
	cT4	2.05	1.08 - 3.91	**0.029**
Pathologic complete response	*No (reference)*			
	Yes	0.35	0.13 - 0.91	**0.032**
Presence of DCIS component	*No (reference)*			
	Yes	1.72	1.09 - 2.7	**0.021**
HER2 status	*Negative (reference)*			
	Positive^b^	0.55	0.31 - 1	**0.048**
Hormonal receptor status ± endocrine therapy	*Positive with endocrine therapy (reference)*			
	Positive without endocrine therapy	1.54	0.46 - 5.13	0.475
	Negative	2.33	1.34 - 4.06	**0.003**
Radiation therapy type	*No radiation therapy (reference)*			
	Chest wall	0.33	0.11 - 0.93	**0.036**
	Chest wall with regional nodes	0.84	0.49 - 1.45	0.531
	Other	1.11	0.18 - 6.88	0.911

Abbreviations: HR = hazard ratio, CI = confidence interval, cT = clinical tumour stage, DCIS = ductal carcinoma in situ, HER2 = human epidermal growth factor receptor 2.

a HR above 1 describes a risk-increasing effect; HR below 1 describes a protective effect.

b ∼97% of HER2-positive patients in the PST group received targeted therapy.

All models showed adequate goodness of fit based on the Cox-Snell residuals, the variable VIFs were below the threshold of 4, and no variable interactions were detected.

All analyses were based on imputed data and yielded results similar to those from the complete cases, with narrower confidence intervals (data not shown).

## 4 Discussion

In this NCR-based study, we analysed the nationwide incidence of and risk factors for isolated LR after mastectomy. LR incidence for guideline-based RT-indication categories was low, as also shown by other studies [19]. Nevertheless, we identified several risk factors for LR for the non-PST and PST groups.

### 4.1 Non-PST group: variables associated with LR

In the non-PST group, the finding that younger age was independently associated with a higher LR risk compared to patients aged 60-69 years (HR 1.43), is consistent with findings from literature that identified an increased LR risk in younger patients, potentially due to more aggressive tumours and/or the presence of an extensive intraductal component[[20], [21], [22]].

Detection through screening, compared to symptomatic detection, was associated with a ±35% reduced LR risk, as it is often associated with detection at an earlier stage and more favourable tumour characteristics [23,24]. Differentiation grade 1, as opposed to grade 2, was associated with reduced LR risk (HR 0.67), and the higher the tumour or nodal stage, the higher the LR risks, which is supported by other studies[[25], [26], [27], [28], [29]].

Sub-localisation in the inner quadrants was associated with higher LR risk (HR 1.36) compared to the outer quadrants. Inner-quadrant tumours have previously been independently associated with poor prognosis for stage I, possibly due to subclinical internal mammary nodal chain involvement [30]. This formed the rationale for the EORTC 22922-10925 trial, which evaluated the benefit of internal mammary irradiation in patients with centrally or medially located tumours [31]. Nonetheless, it is unclear why sub-localisation in inner quadrants is associated with higher LR risk. Raj et al. previously suggested that unintentional underdosing of the breast/chest wall during RT due to heart block or other heart-sparing techniques aimed at reducing the risk of cardiovascular and lung morbidity might contribute to higher LR risk in patients with medially located tumours [32,33].

Our study found that immediate reconstruction in the non-PST group was associated with a 49% higher LR risk, unlike previous smaller studies [34,35]. A potential explanation is that immediate breast reconstruction entails preservation of the breast skin envelope, with or without nipple-areola complex sparing, as in skin-sparing or nipple-sparing mastectomy. This likely preserves the dermal lymphatics and increases the likelihood of residual glandular breast tissue, particularly in nipple-sparing mastectomy[[36], [37], [38]]. Consequently, this may increase the risk of residual tumour cells and/or residual tissue serving as a source of recurrence or new primary tumours, although new primary tumours are likely less reflected in this study given the limited follow-up. In addition, patients in this group were less likely to be treated with postoperative RT compared to those without reconstruction, potentially contributing to LR risk. The Dutch national recommendations for PMRT defined two categories in which shared-decision making (SDM) was recommended: pT1-2N0 with ≥3 risk factors, and pT1-2N1/cT3N0 with ≥1 risk factor. Possibly, the drawbacks of RT for reconstruction more often led to omission of RT in the SDM process than if no reconstruction was performed [39,40]. A few recent retrospective studies support the argument for a surgical mechanism driving these LR. LR was observed in early-stage disease (T1-2N0), for which PMRT is not supported in the guidelines, and was associated with the volume of residual breast tissue after skin- and nipple-sparing mastectomies[[41], [42], [43]]. This raises the concern that residual tumour cells in remaining glandular tissue may drive LR, as they occurred early after surgery [41]. As radiation is associated with an increased risk of complications following breast reconstruction [39,40], its indications should be driven by oncological risks rather than factors that can be improved or reduced by improving surgical technique.

Lastly, targeted treatment for HER2-positive patients (HR 0.3), RT (HR 0.21 for chest wall only and 0.24 for chest wall and regional nodes), and chemotherapy use (HR 0.51) were all associated with a lower LR risk, while no hormonal treatment for hormone-receptor positive patients was associated with a higher LR risk (HR 2.08), which confirms previous studies [27,[43], [44], [45], [46]].

### 4.2 PST group: variables associated with LR

A higher clinical tumour stage was associated with a higher LR risk in the PST group (compared to cT2, HR for cT1 0.90, for cT3 1.36 and for cT4 2.05). While a well-known risk factor for LR, our study demonstrated that it also plays a role in the post-PST setting in mastectomy patients [47]. Pathological complete response was associated with a 65% lower LR risk, supported by studies showing a positive association with disease-free and overall survival after PST[[48], [49], [50]]. The presence of DCIS was associated with a 72% higher LR risk. Similar observations have been reported after BCT, potentially reflecting residual DCIS after PST [20]. A positive HER2 status was also associated with a lower LR risk in the PST group, which can be explained by the fact that ∼97% of these patients received targeted therapy. Though not statistically significant, the absence of hormone therapy for hormone receptor-positive patients was associated with a 54% increased LR risk. Endocrine therapy in these cases was likely not given as per Dutch guidelines, where cN0 oestrogen receptor-positive breast cancer patients with a tumour ≤1 cm or with a grade 1 tumour ≤2 cm are not prescribed endocrine therapy. As we did not have information available on patient preferences or any contraindications, the reason for not having received endocrine therapy in this patient population (n = 188, 3.7%) remains unknown. Lastly, RT was associated with a lower LR risk (HR 0.33 for chest wall only, 0.84 for chest wall and regional nodes).

Overall, at the median follow-up of ∼7 years, LR incidence after mastectomy per indication group was generally low. Although the observational nature of the data limits direct comparison with the risk factors identified in the multivariable analysis, the patterns were generally consistent. In the non-PST group, patients with pT1-2N0 disease and ≥3 risk factors had the highest LR incidence (9.5%). We confirmed that these risk factors – differentiation grade, HR and HER2 receptor status, age, and tumour stage – were associated with LR in our multivariable analysis. In this category, RT was omitted in 66.6% of patients, possibly due to SDM, as the Dutch guidelines from 2008 support SDM for PMRT in this category. In the PST group, patients with cT4 disease had the highest LR incidence (2.5%), consistent with cT4 as an independent risk factor for LR in our multivariable analysis. However, interpretation of these results is limited by the small number of patients and events in the indication groups and by potential deviations from guideline-based indications due to individual treatment decisions in clinical practice.

### 4.3 Relevance for clinical practice

The use of postoperative RT, chemotherapy, endocrine therapy, and HER2-targeted treatments significantly reduced LR risk across both the non-PST and the PST groups. Notably, in the pT1-2N0 subgroup with ≥3 risk factors (differentiation grade 3, triple-negative subtype, age ≤40 years, and tumour size >3 cm), LR incidence rates reached 9.5%: 66% of these patients did not receive RT. Therefore, this identifies a high-risk “early-stage” cohort for which PMRT should be strongly considered. To further substantiate this, a subsequent study is currently evaluating PMRT outcomes in this population to strengthen clinical evidence and refine future consensus recommendations.

### 4.4 Study limitations

The study is based on observational, retrospective data, which limits our ability to control for confounding by severity and residual confounding, as unmeasured patient-related factors may have influenced both treatment selection and outcomes. Consequently, the hazard ratio estimates presented here should be interpreted as associations rather than causal treatment effects [50].

The recurrence data collection has some limitations. Firstly, the algorithm only identified pathologically confirmed LRR diagnoses. A validation study by Van Maaren et al. determined that this resulted in approximately 80% completeness [7]. Therefore, in our study, the LR incidence could have been underestimated. However, as missingness was assumed to be random, we expect it did not influence the observed associations. Secondly, patients with only distant metastases were not identified with this algorithm, which could have affected the correction for competing events. Finally, the smaller sample size of the PST group resulted in less stable estimates in multivariable regression.

There is limited consensus on classifying oestrogen receptor-negative, progesterone receptor-positive tumours as hormone receptor-positive, as they represent a distinct, often more aggressive, subtype [51]. In our cohort, prescribing of endocrine treatment for these tumours was inconsistent, which may have introduced some bias, although the impact is likely limited given their low prevalence in the cohort.

The inclusion of ypTIS within the definition of pCR after PST is debated. Some studies support the inclusion of ypTIS as it is the most used definition in clinical trials and does not reduce prognostic validity [8,52]. Other authors contradict these arguments [50]. Given the lack of consensus, the most widely used definition of pCR, *i.e.* ypT0N0 or ypTISN0, was used to increase comparability with other studies.

## 5 Conclusion

In this study, we showed that overall LR incidence after mastectomy is low and identified several potential risk factors. These findings shed more light on the development of LR after mastectomy. With continued efforts to better understand the patient-, tumour- and treatment-related characteristics associated with LR, despite the observational character of our study, we can incorporate them into personalised treatment before and after mastectomy, and breast cancer care can be further improved.

## Declaration of generative AI and AI-assisted technologies in the manuscript preparation process

During the preparation of this work, the authors used OpenAI's generative artificial intelligence tool ‘ChatGPT’ to assist with debugging and troubleshooting the analysis code. No patient data were provided to the tool, and it was not used to generate or interpret study results. The authors reviewed and edited the output as needed and take full responsibility for the content of the published article.

## Ethical approval

This study has been approved by the privacy committee of the NCR and the NABON-BOOG scientific evaluation committee (reference number K24.014). The study was not required to be assessed under Dutch law on scientific research involving human subjects, as confirmed by the Medical Ethics Committee of the Maastricht Medical Center (METC 2022-3396).

## Funding

This sub-study of the SECRET project was supported by a donation from Mrs. Ruth Levi and Mr. Achiad Gabriel Levi from the Sheba Medical Center in Israel and used for the collaboration with the Netherlands Cancer Registry. The funding body was not involved in the design of the study and collection, analysis, interpretation of data and in writing the manuscript.

## CRediT authorship contribution statement

**T.D.A. Stindt:** Formal analysis, Investigation, Methodology, Visualization, Writing – original draft, Writing – review & editing. **M.C. van Maaren:** Conceptualization, Data curation, Investigation, Methodology, Project administration, Resources, Software, Supervision, Writing – review & editing. **B.V. Offersen:** Writing – review & editing. **S. Siesling:** Resources, Supervision, Writing – review & editing. **D.J.P. van Uden:** Writing – review & editing. **P.M.P. Poortmans:** Writing – review & editing. **L.J. Boersma:** Conceptualization, Writing – review & editing. **O. Kaidar-Person:** Conceptualization, Writing – review & editing.

## Declaration of competing interest

The authors declare the following financial interests/personal relationships which may be considered as potential competing interests: Orit Kaidar-Person reports financial support was provided by Sheba Medical Center. Philip MP Poortmans reports a relationship with Affidea that includes: consulting or advisory. Philip MP Poortmans reports a relationship with MSD that includes: consulting or advisory. Philip MP Poortmans reports a relationship with Sordina Intraoperative Technologies spa that includes: consulting or advisory. PMP Poortmans is specialty editor Radiation Oncology of the Breast. If there are other authors, they declare that they have no known competing financial interests or personal relationships that could have appeared to influence the work reported in this paper.

## Data Availability

Due to privacy regulations, the dataset used in this study is not publicly available. The dataset can only be made available via the NCR (https://iknl.nl/en/ncr/apply-for-data) after a proposal is submitted and approved.
