# Supplementary material for: Factors associated with local breast cancer recurrence after mastectomy in the Netherlands: a retrospective nationwide cohort study
Source: Breast. 2026 Jun 15;88:104844. doi: 10.1016/j.breast.2026.104844 (PMC13284424; doi:10.1016/j.breast.2026.104844)
Supplement: Multimedia component 1 [file mmc1.docx]

# Supplementary Table 1 – Summary of relevant Dutch breast cancer guidelines for 2012

| **Clinical **c**ontext** | **Indications** |
| --- | --- |
| ***Primary systemic therapy (PST)*** |  |
| **Indications for PST** | - Locoregionally extensive breast cancer (Stage III). - Stage II breast cancer when systemic therapy is already indicated at diagnosis and tumour downsizing is desired (e.g., to allow breast-conserving therapy). |
| **Radiotherapy (RT) after PST** | **Locoregional RT (breast/chest wall, axillary, periclavicular):**   - Inoperable local disease (persisting after PST). - Stage III at diagnosis (cT3N1, cT0-2N2-3, cT4). - ypT3N+, ypT4 at surgery. - cN2–3 at diagnosis, or pN2–3 (>3 nodes) after axillary dissection.   **Local RT (breast/chest wall):**   - After BCT. - Tumour-positive resection plane (irradicality). - ypT3, or ypT2 if cT3, when ≥1 of: angioinvasion, grade III, age ≤40 years.   **Parasternal RT:**   - Parasternal metastasis on sentinel node. - Parasternal FDG uptake with anatomic correlate on PET-CT. - Stage III when parasternal drainage is suspected. |
| ***Primary surgery (no PST)*** |  |
| **Adjuvant systemic therapy** | **Positive nodes:**   - Chemotherapy for all patients (unless ≥70 years with HR-negative disease; may still be considered in fit ≥70 patients).   **Negative nodes and unfavourable features:**   - Age ≤35 years (except Grade 1 and ≤1 cm). - Age >35 years and any of:   - Grade 1 tumour ≥2 cm.   - Grade ≥2 tumour ≥1 cm.   - HER2-positive tumour ≥T1b (0.5–1 cm): systemic therapy may be considered. |
| **Radiotherapy after primary surgery** | **Chest Wall RT (after mastectomy):**   - Tumour-positive resection plane / irradicality (cT4, pT4). - pT3, when ≥1 of: angioinvasion, grade III, age ≤40 years.   **Chest Wall RT (consider when):**   - pT1-2pN1 and one or more of: angioinvasion, grade III, age ≤40 years, tumour >3 cm. - pT1-2pN0 and ≥3 of: angioinvasion, grade III, age ≤40 years, tumour >3 cm.   **Locoregional RT (BCT or mastectomy):**   - ≥4 positive nodes. - Tumour-positive axillary apex. |
| **Radiotherapy dose recommendations** | From the Dutch Platform for Radiotherapy of Breast Cancer   - Elective dose only: 15 × 2.66 Gy. - Low boost: 21 × 2.66 Gy (tumour bed) and 21 × 2.17 Gy (remaining volume). - High boost: 23 × 2.66 Gy (tumour bed) and 21 × 2.03 Gy (remaining volume). |
