# Supplementary material for: Factors associated with local breast cancer recurrence after mastectomy in the Netherlands: a retrospective nationwide cohort study
Source: Breast. 2026 Jun 15;88:104844. doi: 10.1016/j.breast.2026.104844 (PMC13284424; doi:10.1016/j.breast.2026.104844)
Supplement: Multimedia component 2 [file mmc2.docx]

# Supplementary Table 2 – Risk factors for post-mastectomy LR in non-PST patients based on the full multivariable Cox regression model of the imputed dataset.

| Factor | Level | HR^i^ | 95% CI | P-value |
| --- | --- | --- | --- | --- |
| Age | *60-69 (reference)* | | | |
|  | <40 | 1.44 | 0.75 - 2.78 | 0.277 |
|  | 40-49 | 0.96 | 0.56 - 1.65 | 0.895 |
|  | 50-59 | 1.47 | 1.01 - 2.13 | **0.042** |
|  | 70-79 | 1.17 | 0.82 - 1.67 | 0.384 |
|  | >79 | 0.9 | 0.6 - 1.33 | 0.592 |
| Menopausal status | *Post (reference)* | | | |
|  | Pre | 1.03 | 0.68 - 1.57 | 0.873 |
|  | Peri | 0.54 | 0.27 - 1.08 | 0.082 |
| Screening | *No (reference)* | | | |
|  | Yes | 0.66 | 0.49 - 0.88 | **0.005** |
| Sublocalisation | *Outer quadrants (reference)* | | | |
|  | Inner quadrants | 1.36 | 1.02 - 1.81 | **0.034** |
|  | Central parts | 0.92 | 0.62 - 1.38 | 0.695 |
|  | Overlapping lesions | 1.06 | 0.81 - 1.38 | 0.69 |
| Morphology | *Ductal (reference)* | | | |
|  | Lobular | 1.15 | 0.83 - 1.6 | 0.402 |
|  | Mixed ductal lobular | 0.76 | 0.4 - 1.44 | 0.399 |
|  | Other | 0.67 | 0.38 - 1.19 | 0.17 |
| Differentiation grade | *Grade 2 (reference)* | | | |
|  | Grade 1 | 0.68 | 0.49 - 0.96 | **0.026** |
|  | Gade 3 | 1.06 | 0.8 - 1.41 | 0.683 |
| Multifocality | *No (reference)* | | | |
|  | Yes | 1.1 | 0.85 - 1.42 | 0.481 |
| pT | *pT1 (reference)* | | | |
|  | pT2 | 1.45 | 1.11 - 1.91 | **0.007** |
|  | pT3 | 1.91 | 1.17 - 3.11 | **0.01** |
|  | pT4 | 2.1 | 0.97 - 4.55 | 0.059 |
| pN | *pN0 (reference)* | | | |
|  | pN1 | 1.45 | 1.11 - 1.9 | **0.007** |
|  | pN2 | 2.33 | 1.4 - 3.89 | **0.001** |
|  | pN3 | 3.34 | 1.89 - 5.89 | **<0.001** |
| Presence of DCIS component | *No (reference)* | | | |
|  | Yes | 0.97 | 0.76 - 1.23 | 0,801 |
| Immediate reconstruction | *No (reference)* | | | |
|  | Yes | 1.49 | 1.1 - 2.01 | **0.009** |
| Hormonal receptor status ± endocrine therapy | *Positive with endocrine therapy (reference)* | | | |
|  | Positive without endocrine therapy | 2.12 | 1.59 - 2.84 | **<0.001** |
|  | Negative | 2.85 | 2.09 - 3.89 | **<0.001** |
| HER2 status ± targeted therapy | *Negative (reference)* | | | |
|  | Positive with targeted therapy | 0.3 | 0.15 - 0.6 | **0.001** |
|  | Positive without targeted therapy | 0.87 | 0.56 - 1.35 | 0.529 |
| Radiation therapy type | *No radiation therapy (reference)* | | | |
|  | Chest wall | 0.21 | 0.1 - 0.43 | **<0.001** |
|  | Chest wall with regional nodes | 0.24 | 0.15 - 0.39 | **<0.001** |
|  | Other | 0.73 | 0.3 - 1.81 | 0.5 |
| Chemotherapy | *No (reference)* | | | |
|  | Yes, post-surgical | 0.51 | 0.37 - 0.72 | **<0.001** |

^i^ HR above 1 describes a risk-increasing effect; HR below 1 describes a protective effect.

Abbreviations: HR = hazard ratio, CI = confidence interval, pN = pathological nodal stage, pT = pathological tumour stage, DCIS = ductal carcinoma in situ, HER2 = human epidermal growth factor receptor 2.
