# Supplementary material for: Factors associated with local breast cancer recurrence after mastectomy in the Netherlands: a retrospective nationwide cohort study
Source: Breast. 2026 Jun 15;88:104844. doi: 10.1016/j.breast.2026.104844 (PMC13284424; doi:10.1016/j.breast.2026.104844)
Supplement: Multimedia component 3 [file mmc3.docx]

# Supplementary Table 3 – Risk factors for post-mastectomy LR in PST patients based on the full multivariable Cox regression model of the imputed dataset.

| Factor | Level | HR^i^ | 95% CI | P-value |
| --- | --- | --- | --- | --- |
| Menopausal status | *Post (reference)* | | | |
|  | Pre | 0.84 | 0.48 - 1.48 | 0.55 |
|  | Peri | 1.46 | 0.68 - 3.14 | 0.328 |
| Screening | *No (reference)* | | | |
|  | Yes | 0.45 | 0.16 - 1.31 | 0.142 |
| Sublocalisation | *Outer quadrants (reference)* | | | |
|  | Inner quadrants | 0.93 | 0.41 - 2.12 | 0.857 |
|  | Central parts | 1.34 | 0.56 - 3.19 | 0.506 |
|  | Overlapping lesions | 1.56 | 0.91 - 2.66 | 0.101 |
| Morphology | *Ductal (reference)* | | | |
|  | Lobular | 0.69 | 0.27 - 1.74 | 0.419 |
|  | Mixed ductal lobular | 0.96 | 0.22 - 4.15 | 0.951 |
|  | Other | 0.69 | 0.16 - 2.98 | 0.616 |
| Differentiation grade | *Grade 2 (reference)* | | | |
|  | Grade 1 | 0.9 | 0.35 - 2.36 | 0.831 |
|  | Gade 3 | 1.55 | 0.85 - 2.82 | 0.148 |
| Multifocality | *No (reference)* | | | |
|  | Yes | 0.9 | 0.55 - 1.49 | 0.688 |
| cT | *cT2 (reference)* | | | |
|  | cT1 | 0.96 | 0.41 - 2.23 | 0.914 |
|  | cT3 | 1.35 | 0.75 - 2.42 | 0.308 |
|  | cT4 | 2.06 | 1.03 - 4.11 | **0.04** |
| cN | *cN1 (reference)* | | | |
|  | cN0 | 1.16 | 0.64 - 2.08 | 0.615 |
|  | >cN1 | 0.43 | 0.18 - 1.05 | 0.064 |
| Pathologic complete response | *No (reference)* | | | |
|  | Yes | 0.35 | 0.13 - 0.94 | **0.038** |
| Presence of DCIS component | *No (reference)* | | | |
|  | Yes | 1.62 | 1.01 - 2.62 | **0.046** |
| HER2 status | *Negative (reference)* | | | |
|  | Positive | 0.53 | 0.29 - 0.98 | **0.042** |
| Immediate reconstruction | *No (reference)* | | | |
|  | Yes | 1.01 | 0.57 - 1.78 | 0.981 |
| Hormonal receptor status ± endocrine therapy | *Positive with endocrine therapy (reference)* | | | |
|  | Positive without endocrine therapy | 1.64 | 0.48 - 5.57 | 0.417 |
|  | Negative | 2.3 | 1.29 - 4.09 | **0.006** |
| Radiation therapy type | *No radiation therapy (reference)* | | | |
|  | Chest wall | 0.32 | 0.11 - 0.95 | **0.041** |
|  | Chest wall with regional nodes | 0.95 | 0.5 - 1.82 | 0.873 |
|  | Other | 1.16 | 0.18 - 7.43 | 0.873 |
| Chemotherapy | *Yes, pre-surgical (reference)* | | | |
|  | No | 0.73 | 0.24 - 2.25 | 0.578 |
|  | Yes, post-surgical | 2.41 | 0.31 - 18.98 | 0.394 |
|  | Yes, pre- and post-surgical | 1.26 | 0.52 - 3.05 | 0.599 |

^i^ HR above 1 describes a risk-increasing effect; HR below 1 describes a protective effect.

Abbreviations: HR = hazard ratio, CI = confidence interval, cT = clinical tumour stage, cN = clinical nodal stage, DCIS = ductal carcinoma in situ, HER2 = human epidermal growth factor receptor 2.
